# Classical Active Ingredients and Extracts of Chinese Herbal Medicines: Pharmacokinetics, Pharmacodynamics, and Molecular Mechanisms for Ischemic Stroke

**DOI:** 10.1155/2021/8868941

**Published:** 2021-03-13

**Authors:** Ting Zhu, Lei Wang, Yicheng Feng, Guibo Sun, Xiaobo Sun

**Affiliations:** ^1^Beijing Key Laboratory of Innovative Drug Discovery of Traditional Chinese Medicine (Natural Medicine) and Translational Medicine, Institute of Medicinal Plant Development, Peking Union Medical College and Chinese Academy of Medical Sciences, Beijing 100193, China; ^2^Key Laboratory of Bioactive Substances and Resources Utilization of Chinese Herbal Medicine, Ministry of Education, Institute of Medicinal Plant Development, Chinese Academy of Medical Sciences & Peking Union Medical College, Beijing 100193, China; ^3^Key Laboratory of New Drug Discovery Based on Classic Chinese Medicine Prescription, Chinese Academy of Medical Sciences, Beijing 100193, China; ^4^Harbin University of Commerce, Heilongjiang 150000, China; ^5^Beijing University of Chemical Technology, Beijing 100029, China

## Abstract

Stroke is a leading cause of death and disability worldwide, and approximately 87% of cases are attributed to ischemia. The main factors that cause ischemic stroke include excitotoxicity, energy metabolism disorder, Ca^+^ overload, oxidative damage, apoptosis, autophagy, and inflammation. However, no effective drug is currently available for the comprehensive treatment of ischemic stroke in clinical applications; thus, there is an urgent need to find and develop comprehensive and effective drugs to treat postischemic stroke. Traditional Chinese medicine (TCM) has unique advantages in treating ischemic stroke, with overall regulatory effects at multiple levels and on multiple targets. Many researchers have studied the effective components of TCMs and have achieved undeniable results. This paper reviews studies on the anticerebral ischemia effects of TCM monomers such as tetramethylpyrazine (TMP), dl-3-n-butylphthalide (NBP), ginsenoside Rg1 (Rg1), tanshinone IIA (TSA), gastrodin (Gas), and baicalin (BA) as well as effective extracts such as *Ginkgo biloba* extract (EGB). Research on the anticerebral ischemia effects of TCMs has focused mostly on their antioxidative stress, antiapoptotic, anti-inflammatory, proangiogenic, and proneurogenic effects. However, the research on the use of TCM to treat ischemic stroke remains incompletely characterized. Thus, we summarized and considered this topic from the perspective of pharmacokinetics, pharmacological effects, and mechanistic research, and we have provided a reference basis for future research and development on anticerebral ischemia TCM drugs.

## 1. Introduction

Stroke is a leading cause of death and disability worldwide, and approximately 87% of cases are attributed to ischemia [1]. The risk associated with ischemic stroke results mainly from cerebral ischemia/reperfusion (I/R) injury, a pathological condition characterized by an initial block of the cerebral blood flow supply to an organ followed by the restoration of perfusion and reoxygenation [2–4]. In addition, I/R injury can cause disease under various adverse conditions, primarily relating to blood-brain barrier (BBB) leakage, energy metabolism disorder, excitotoxicity, Ca^2+^ overload, aberrant mitochondrial responses, oxidative stress, and autophagy and aberrant immune responses [5, 6]. Moreover, these factors and mechanisms are interrelated and interact with each other, resulting in apoptosis or neuronecrosis in the ischemic area [7, 8].

Currently, thrombolysis and thrombectomy are effective treatments for acute ischemic stroke. However, due to time window constraints, these methods can only be used in a minority of patients [9, 10]. Another effective treatment for acute ischemic stroke is neuroprotective drugs, which primarily include calcium channel antagonists, free radical scavengers, glutamate antagonists, and cell membrane stabilizers [11]. However, because most therapeutic targets are identified by experiments on the molecular mechanism of cerebral ischemia in animals rather than humans, the application of neuroprotective drugs has failed in the clinic [12]. Therefore, widely applicable therapeutic approaches for ischemic stroke are urgently needed.

Traditional Chinese medicine (TCM) has some prominent advantages in the comprehensive treatment of multisite, multitarget conditions and in overall regulation [13]. Some TCMs, such as *Panax notoginseng* [14], *Ginkgo biloba* [15], *Rehmannia glutinosa* [16], and *Gastrodia elata* [17], have better therapeutic effects on neurological disorders than on other disorders. The research and development of neuroprotective drugs based on the TCM components tetramethylpyrazine (TMP), dl-3-n-butylphthalide (NBP), and *ginkgo biloba* extract (EGB) have shown that these compounds also have neuroprotective activity in vivo and in vitro and an extensive basis for clinical application. Here, we conduct a systematic review of all the available studies to analyze and summarize the pharmacokinetics, pharmacological effects, and mechanisms of TCM in experimental studies on ischemic stroke.

To explore and summarize the pharmacokinetics, pharmacological effects, and relevant mechanisms of these TCM components in ischemic stroke systematically, we conducted this review by searching the PubMed database using a drug name, such as “Tetramethylpyrazine”, and “Stroke” as search terms to obtain the literature. This approach allowed us to summarize and analyze the literature on the pharmacokinetics, pharmacological effects, and possible mechanisms of active TCM components in ischemic stroke, provide references for the research and application of TCM in neuroprotection, and further provide additional citation-based information for the development of candidate drugs that can be applied during clinical trials on strokes.

## 2. Chinese Herbal Monomers

### 2.1. Tetramethylpyrazine (TMP)

**Figure 1:**
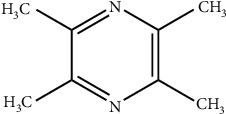
Chemical structural formula of TMP.

Chuanxiong is a crude herbal drug derived from the dried roots or rhizomes of *Rhizoma Chuanxiong* and has long been used in China to treat cardiovascular and cerebrovascular diseases [18–20]. To date, more than 30 compounds have been isolated, and they are derived from three main chemical groups: alkaloids, phenolic acids, and phthalates [18]. TMP is an active alkaloid monomer that was isolated from the rhizome of the Chinese herb *Rhizoma Chuanxiong* (see Figure 1 for the chemical structure) [21], and the evidence has shown that its pharmacological activities are notorious for including neuroprotective effects against cerebral ischemia [22, 23]. Drugs consisting of TMP preparations, such as TMP phosphate tablets, TMP phosphate injections, and TMP hydrochloride injections, have been verified clinically and experimentally [24, 25].

#### 2.1.1. Pharmacokinetics of TMP

The pharmacokinetic study of TMP gradually developed in the middle and late 1980s. Early literature reported the pharmacokinetic parameters of TMP in mice, rats, and dogs. After 3H-TMP is injected into the tail vein of mice, it is mainly distributed in the liver, bile, small intestine, brain, kidney, and other organs, especially the liver is the most obvious, indicating that the liver is an important target organ sensitive to drugs, and is ultimately excreted from the urine through the kidneys in vitro [26]. Rats were intragastrically administered with TMP 0.2 g·kg^−1^. After absorption, the drug can be distributed in various tissues. After 30 minutes of taking the drug, the content in the liver is the highest, followed by the kidneys, and the brain is the third. At different times after administration, there is a small amount of TMP in the large intestine, and a small amount may be excreted in the feces [27]. Most of the drugs were absorbed rapidly in the gastrointestinal tract, distributed widely, and eliminated quickly after the dogs were given TMP intragastrically. It can cross the BBB and is eventually excreted through the kidneys [28]. After intramuscular injection of 40 mg of TMP in normal volunteers, the in vivo process conformed to the one-compartment model. *T*_1/2_ is 27.5 min, and the apparent distribution volume (*V*_*d*_) is 1.33 L·kg^−1^. The drugs are mainly distributed in organs such as the liver, gallbladder, small intestine, brain, and kidney, especially the liver is the most obvious, indicating that the liver is the target organ [29].

In recent years, a comparative study on the pharmacokinetics of TMP monomer and compatibility has been reported in the literature. Hu et al. studied the pharmacokinetics of TMP combined with borneol (BN) in a microemulsion-based transdermal therapeutic system (TMP-BN-ME-TTS) in male SD rats. The results showed that the AUC of TMP in the brain increased by 60% in TMP-BN-ME-TTS group, indicating that BN promoted the distribution of TMP in the brain [30]. In addition, intranasal delivery can bypass the BBB and deliver the drug directly to the brain. However, intrinsic drug distribution to the brain after intranasal administration is not enough to achieve the desired clinical efficacy. Gao et al. studied the feasibility of using polysorbate 80 as an absorption enhancer to increase the distribution of drugs in the brain, taking tetramethylpyrazine phosphate (TMPP) as a model drug. The results showed that intranasal administration could significantly improve the brain targeting efficiency of TMPP. Upon intranasal administration, the addition of polysorbate 80 could significantly increase the TMPP concentration in both plasma and brain and showed a linear relationship [31].

#### 2.1.2. Anti-Ischemic Effects and Mechanisms of TMP

TMP is considered one of the main bioactive compounds responsible for the pharmaceutical activity of Chuanxiong.

TMP phosphate for clinical injection is given by intravenous drip of 100 mg each time. In rats, 20 mg·kg^−1^ (twice the clinical equivalent dose) TMP phosphate was usually injected intraperitoneally to evaluate its protective effect on ischemic stroke. In addition, the evidence has shown that TMP can reduce neurological functional loss, promote neurogenesis and oligodendrogenesis [32], enhance neuronal dendritic plasticity [33], decrease cerebral edema and BBB permeability [34], and inhibit macrophage/microglia activation [22]. Additionally, an increasing amount of evidence indicates that TMP exerts anticerebral ischemia effects both in vivo and in vitro. Various mechanisms have been suggested to underlie the activities of TMP, including the activation of free radical scavenging [35, 36], the inhibition of Ca^2+^ overload [35], the maintenance of mitochondrial function [35], the suppression of apoptosis [37, 38] and inflammation [23, 39], and the stimulation of neuronal differentiation [40]. Current in vivo results have shown that treating with TMP can significantly reduce brain infarction, behavioral functional impairment, and cerebral water content [32, 33, 35, 39, 41] in rats with middle cerebral artery occlusion- (MCAO-) induced cerebral ischemia. Furthermore, TMP obviously promotes neurogenesis [32, 40] and oligodendrogenesis [32], enhances dendritic plasticity [33], attenuates BBB disruption [34, 41, 42], and inhibits neuroinflammation [22, 39].

First, TMP administered at different doses (20 and 40 mg·kg^−1^ body mass, daily) for 7 d and 21 d for enhanced neural progenitor/precursor cell (NPC) migration towards the ischemic region in rats by activating the phosphatidylinositol 3-kinase (PI3K) pathway [40]. In addition, treating with TMP nitrone (a novel nitrone derivative of TMP) via intravenous injection through the tail vein (30 mg·kg^−1^ body mass, twice daily with a 6 h interval) for a total of 7 d significantly reduced cerebral infarction, preserved and/or restored neurological function, and promoted neurogenesis and oligodendrogenesis in rats after MCAO. This mechanism works through the activation of the AKT/cAMP-responsive element-binding (CREB) protein by increasing brain-derived neurotrophic factor (BDNF) expression. These effects demonstrated that TMP nitrone promotes neuronal regeneration after ischemic stroke via the upregulation of AKT/CREB by increasing BDNF expression [32].

Second, rats that received an intraperitoneal injection of 20 mg·kg^−1^ TMP 15 min before the onset of ischemia exhibited lower neurological scores and levels of brain infarction and edema than untreated rats. These effects were accompanied by decreased BBB permeability and increased levels of occludin and claudin-5, two tight junction protein components of the BBB [41]. Intravenous injection of a novel analog of TMP, TMP-2′-O-sodium ferulate (10.8, 18 and 30 mg·kg^−1^ body mass, daily), for 2 d significantly reduced the cerebral water content, improved the BBB permeability, and decreased the matrix metalloproteinase-9 (MMP-9) and aquaporin 4 (AQP4) levels [42]. In addition, after 12 h, the intraperitoneal injection of TMP (40 mg·kg^−1^ body mass) reduced neurological functional loss, decreased cerebral edema and BBB permeability, and increased the expression of tight junction proteins by inhibiting the JAK/STAT signaling pathway [34].

Third, TMP (20 mg·kg^−1^ body mass) was injected intraperitoneally two times, at 30 min before and 60 min after occlusion, and was shown to reduce neuronal loss, macrophage/microglia activation, neutrophil infiltration into the brain parenchyma, circulating neutrophils, endothelial adhesion, spontaneous nitric oxide (NO) production, and stimulus-activated NO production after cerebral ischemia. The mechanism was shown to be related to the elevation of nuclear factor erythroid 2-related factor (Nrf2)/HO-1 expression and the inhibition of HMGB1/TLR4, AKT, and extracellular signal-regulated kinase (ERK) signaling [22]. Importantly, the intravenous injection of TSF (10.8, 18, and 30 mg·kg^−1^ body mass, daily) for 72 h significantly improved neurological deficits and reduced the brain water content and infarct size, which was accompanied by a decrease in the concentration of several proinflammatory cytokines (TNF-*α*, IL-1*β*, MCP-1, CD11b, ICAM-1, and inducible nitric oxide synthase (iNOS)) and an increase in the concentration of an anti-inflammatory cytokine (IL-10), all of which are involved in inhibiting of the TLR-4/NF-*κ*B signaling pathway. These effects indicate that TMP exerts a neuroprotective effect against ischemic stroke that might be mediated through the suppression of these inflammatory pathways [22, 39].

In vitro pharmacological studies indicated that TMP may also promote NPC migration [32, 40] and alleviate the inflammatory response [23]. In addition, TMP exhibited significant activities, inhibiting apoptosis, scavenging free radicals, blocking calcium overload, and maintaining mitochondrial function.

Brain microvascular endothelial cells (BMECs) are indispensable components of the BBB, and protecting BMECs against oxygen-glucose deprivation (OGD) is important when treating ischemic stroke [36]. Treating with TMP at different concentrations (14.3, 28.6, and 57.3 *μ*M) was shown to reduce the apoptosis rate significantly in a concentration-dependent manner and to downregulate the key proteins in the Rho/Rho kinase (ROCK) signaling pathway notably [36]. TMP (200 *μ*g·mL^−1^) also significantly reduced the apoptosis rate of anoxia/reoxygenation- (A/R-) induced primary hippocampal neurons, which was accompanied by decreased mRNA levels of the JNK kinases MKK4 and MKK7 as well as decreased protein levels of C-fos, C-jun, and P-JNK [38].

TMP exhibits potent antioxidant activity, as reflected mainly by its ability to inhibit intracellular reactive oxygen species (ROS) generation. Bone marrow-derived mesenchymal stem cells (BMSCs) pretreated with TMP (10, 25, 50, 100, and 200 *μ*mol·L^−1^) for 24 h and then exposed to 500 *μ*mol·L^−1^ H_2_O_2_ for 24 h exhibited significantly increased viability and decreased apoptosis and ROS generation. Furthermore, the protective effects of TMP were related to increased Bcl-2 expression, attenuated Bax expression, and enhanced levels of phosphorylated AKT (p-AKT) and p-ERK1/2 [37].

OGD-induced primary cortical neuron models are used to mimic cerebral I/R injury in animals and are extensively used in ischemic stroke studies. TMP nitrone (30, 100, and 300 *μ*M) was proven to increase the number of viable neurons significantly, decrease apoptosis, quench the overproduction of intracellular free radicals, inhibit Ca^2+^ overload, and maintain mitochondrial function in a concentration-dependent manner [35].

In summary, as shown in Table 1, TMP can promote neuronal differentiation and migration and enhance dendritic plasticity, and these effects are closely associated with the promotion of neurogenesis and oligodendrogenesis, the activation of the BDNF/AKT/CREB pathway [32], and increases in PI3K/AKT, protein kinase C (PKC), and ERK expression [40]. TMP can also decrease cerebral edema and BBB permeability, increase tight junction protein expression and suppress MMP-9 and AQP4 expression [34, 41, 42] via a mechanism related to inhibiting JAK/STAT signaling pathway activation [34]. Additionally, treating with TMP enhances protection against cerebral ischemic injury via antiapoptotic, antioxidant, and anti-inflammatory effects. Several processes are involved: TMP reduces the expression of proinflammatory cytokines (TNF-*α*, IL-1*β*, MCP-1, CD11b, ICAM-1, and iNOS) [39], decreases the level of the apoptotic factors Bax and caspase-3 [35, 36], and inhibits ROS generation [36, 37]. These mechanisms might be associated with the ability to activate the PI3K/AKT/p-GSK3*β* cell survival pathway [35]; elevate Nrf2/HO-1 expression and inhibit HMGB1/TLR4, AKT, and ERK signaling [22]; inhibit the P38 MAPK and NF-*κ*B signaling pathways [23]; inhibit the Rho/ROCK signaling pathway [36]; regulate the PI3K/AKT and ERK1/2 signaling pathways [37]; and inhibit the JNK signaling pathway.

### 2.2. DL-3-N-Butylphthalide (NBP)

**Figure 2:**
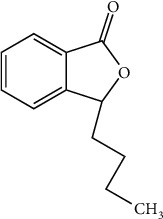
Chemical structural formula of NBP.

L-3-n-butylphthalide is the primary medicinal ingredient of the TCMs Chuanxiong, bergamot, and angelica, and it was originally extracted from the seeds of *Apium graveolens* Linn. Then, a racemic mixture, dl-3-n-butylphthalide (NBP), was artificially synthesized and became the first new drug with independent intellectual property rights for use in cerebrovascular disease treatment in China [44] (Figure 2).

#### 2.2.1. Pharmacokinetics of NBP

Although the pharmacological properties of NBP are widely studied, the pharmacokinetics of NBP is not well understood. Only a few studies have explored its metabolism. Diao et al. identified 23 metabolites from human plasma and urine after oral administration of 200 mg NBP. The results showed that NBP is well absorbed after oral administration and undergoes extensive metabolism to produce a variety of oxidized and conjugated metabolites. Hydroxylation is the primary pathway of metabolism, which mainly occurs on the *n*-butyl side chain. Renal excretion is the primary elimination pathway. In vitro studies demonstrated that cytochrome P450s (P450), alcohol dehydrogenase (ADH), and aldehyde dehydrogenase (ALDH) enzymes are all involved in the overall clearance of NBP [45]. Potassium 2-(1-hydroxypentyl)-benzoate (PHPB) is a new prodrug of NBP that is used to treat ischemic stroke. Li et al. studied the pharmacokinetics of PHPB. In vivo studies have shown that PHPB can be quickly and completely converted into NBP mediated by paraoxonase in fat, brain, and stomach [46]. The oral AUC value of NBP converted from PHPB was two- to three-fold greater than those from direct NBP administration.

#### 2.2.2. Anti-Ischemic Effects and Mechanisms of NBP

NBP soft capsules for clinical application are given by oral administration of 200 mg each time. In rats, 20 mg·kg^−1^ (clinical equivalent dose) NBP soft capsules were usually injected intraperitoneally to evaluate its protective effect on ischemic stroke. NBP has many pharmacological activities, such as protecting the BBB [47–49], enhancing hemodynamics and cerebral blood flow [50], inhibiting platelet activation [51], and suppressing neurovascular inflammation [48]. Importantly, NBP can also improve ATP metabolism [52], increase synaptic growth [53, 54], and promote angiogenesis [55, 56], remyelination [57], and neurogenesis [53].

The diameter of the axon and the thickness and spacing of myelin determine the rate of neuronal conduction along an axon. Oligodendrocyte precursor cells (OPCs) generate myelin-forming oligodendrocytes, which are essential for myelin regeneration and functional recovery after cerebral ischemia [58–60]. NBP (70 mg·kg^−1^ body mass, daily) was administered by oral gavage for 2 weeks, and it significantly promoted the differentiation and maturation of OPCs in the perilesional white matter, enhanced the length of crossing corticospinal tract (CST) fiber growth into the denervated hemispheres, elevated the expression of synapse-associated proteins, including PSD95 and VGlut-1, and promoted neurogenesis. These effects were associated with increasing BDNF expression and reducing neurite outgrowth inhibitor (NogoA) expression in the perilesional area [53, 57]. In addition, NBP was found to promote dendrite development by inactivating PI3K/AKT signaling in cortical neurons that had been subjected to OGD injury [54]. These results highlight the effects of NBP on the growth and remyelination of synapses and reveal the therapeutic potential of this compound in cerebral ischemia.

After cerebral ischemia, angiogenesis increases regional cerebral blood flow (rCBF) and collateral vessel circulation, which are positively related to the survival and recovery of stroke patients and laboratory animals [61]. Treating with NBP (80 mg·kg^−1^ body mass, daily) by oral gavage for 14 d was found to increase the number of cluster of differentiation 31^+^ (CD31^+^) microvessels, the number of CD31^+^/bromodeoxyuridine^+^ (BrdU^+^) proliferating endothelial cells, and the functional vascular density significantly and to promote the expression of vascular endothelial growth factor (VEGF) and angiopoietin-1. These effects were associated with increased sonic hedgehog expression after NBP treatment [55]. In addition, intraperitoneal injections of NBP (6.5 mg·kg^−1^ body mass, twice daily) for 14 d increased the white matter integrity, microvessel formation, and the level of the tight junction protein occludin by promoting the expression of hypoxia-induced factor-1*α* (HIF-1*α*), VEGF, Notch, and delta-like ligand 4 (DLL-4, 62). The activation of cellular endothelial nitric oxide synthase (eNOS) is crucial for its protective effect on vascular proliferation. NBP can increase the expression of PGC-1*α* during OGD in a way that is dependent on the eNOS activity [56]. Therefore, NBP may contribute to vascular proliferation after a stroke.

Accumulating evidence has demonstrated that small molecule metabolites and alterations in their metabolic pathways play an important role in ischemic stroke development [62]. Intraperitoneal injections of NBP (20 mg·kg^−1^ body mass, daily) for 7 d significantly improved ATP metabolism, antioxidant levels, and the sodium-potassium ion balance in a rat model of permanent MCAO (pMCAO) according to matrix-assisted laser desorption ionization time-of-flight mass spectrometry (MALDI-TOF-MS) imaging [63]. Hence, NBP exerts anti-ischemic effects through the regulation of small molecule metabolism in the brain.

These experimental results reveal that NBP exerts anti-ischemic effects both in vivo and in vitro, partially by improving ATP metabolism [63], promoting angiogenesis [55, 56], stimulating synaptic growth [53, 54], and promoting remyelination [57] and neurogenesis [53], as well as by upregulating sonic hedgehog expression [55], increasing endothelial PGC-1*α* expression by regulating eNOS activity [56], inactivating PI3K/AKT signaling [54], increasing BDNF expression, and reducing NogoA expression in the perilesional area [57] (Table 2).

### 2.3. Ginsenoside Rg1 (Rg1)

**Figure 3:**
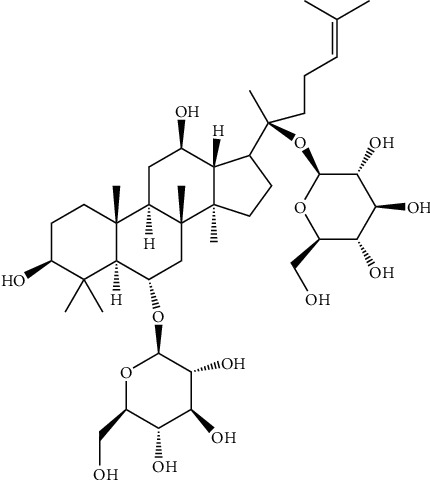
Chemical structural formula of Rg1.

*P. notoginseng* (Burk) F. H. Chen and *P. ginseng* C. A. Mey, two of the most widely used Chinese medicinal herbs, have several hundred years of medicinal history in treating cardiovascular disease in Asia [65]. Saponins are a primary bioactive component of *P. notoginseng* [66, 67] and *P. ginseng* [68, 69], and they exhibit multiple pharmacological activities, such as anticoagulant [70], anti-inflammatory [71], antioxidant [72, 73], and antiapoptotic [74] activities. With the development of advanced technology, more than 80 types of monomeric saponins have been isolated and identified from the different parts of *P. notoginseng* (roots, stems, leaves, and flower heads) [75]. Rg1 (see Figure 3 for the chemical structure) is considered one of the primary active components that is primarily isolated from the roots or stems of *P. notoginseng* and *P. ginseng* and chemically belongs to the 20(S)-protopanaxatriol saponin group [76].

#### 2.3.1. Pharmacokinetics of Rg1

The absorption of PNS varies with the changes of environmental conditions, such as position, different formulations, and different routes of administration [77]. Feng et al. studied the absorption of Rg1 in the whole intestinal segment of rats. The results found that the absorption of Rg1 in the stomach, duodenum, jejunum, and ileum are 0.0552, 0.112, 0.065, and 0.0555 h^−1^, respectively, indicating that Rg1 can be absorbed in the whole intestinal segment, with the highest absorption in the duodenum and the lowest in the stomach [78]. Liang et al. showed that carbomer and borneol are suitable absorption promoters, which can significantly increase the absorptive rate of PNS by increasing the permeability of Rg1 in the intestinal wall of rats [79]. Li et al. studied the pharmacokinetics of Nao-Qing microemulsion in rats by intranasal or intragastric routes. Rat brain tissues were collected at predetermined time intervals, and the contents of Rg1 were analyzed by high-performance liquid chromatography (HPLC). The results showed that the AUC_0−∞_, half-life for distribution (*t*_1/2*α*_), and half-life for elimination (*t*_1/2*β*_) for Rg1 in the brain for intranasal administration were 5.83, 0.26, and 7.10 times those for intragastric administration [80]. The data indicated that intranasal administration can promote the absorption of drugs in Nao-Qing microemulsion and achieve fast effect. Xue et al. studied the pharmacokinetics of Rg1 in the medial prefrontal cortex (mPFC), hippocampus (HIP), and lateral ventricle (LV) of rats after subcutaneous injection. The results showed that the elimination of Rg1 in the mPFC group was significantly slower than that in the HIP and LV groups, and the AUC was significantly higher than that in the HIP group [81]. Feng et al. clarified that Rg1 can be easily distributed to most tissues. However, it could not effectively cross the BBB [82]. In order to develop a drug delivery system across the BBB, Shen et al. prepared a poly-*γ*-glutamic acid (*γ*-PGA)-based nanoparticles (PHRO) to load Rg1 and conjugated with OX26 monoclonal antibody. In vitro experiments showed that PHRO promoted angiogenesis and crossed the BBB. In vivo experiments showed that PHRO could increase the brain distribution of Rg1 in diabetic cerebral infarction group, and the improvement of cerebral necrosis in rats in diabetic cerebral infarction group was significantly stronger than that in free Rg1 group [83], indicating that the drug delivery system provides more opportunities for drugs to penetrate the BBB.

**Figure 4:**
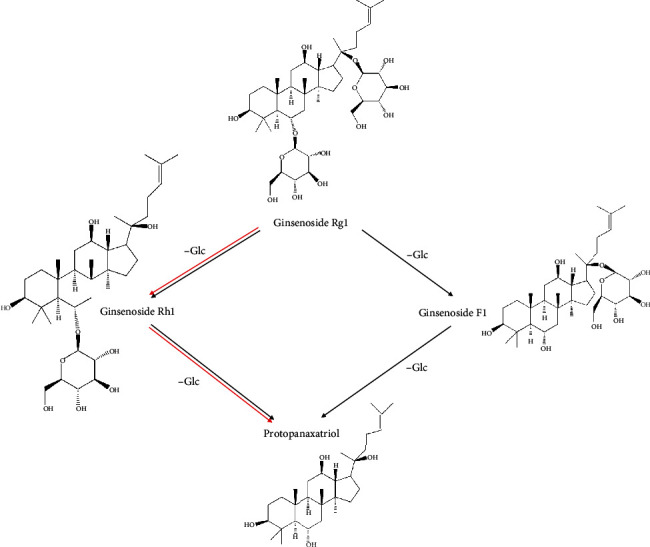
Metabolites of Rg1 in the intestine. The black arrows indicate the metabolic route in the rat intestine, while the red arrows indicate the metabolic route in the human intestine.

The metabolic pathway of Rg1 mainly involves the hydrolyzation of the 6- and 20-glucoside bond, which is widely metabolized in the intestinal tract. The process is catalyzed by *β*-glucosidase which is excreted by intestinal flora [77]. With different types of intestinal bacteria, the metabolic pathways between rats and humans are also different. In rat intestinal tract, Rg1→Rh1 and F1→20(S)-protopanaxatriol [82]. In human intestinal tract, Rg1→Rh1→20(S)-protopanaxatriol [84]. The metabolic route of Rg1 is listed in Figure 4.

Feng et al. studied the pharmacokinetics of Rg1 by measuring the concentration of Rg1 in bile, urine, and feces of rats. The results showed that after intravenous injection, the mean recovery of Rg1 in bile, urine, and feces was 60.77%, 27.95%, and 7.64%, respectively. Rg1 was completely excreted within 8 h in bile and 12 h in urine and feces, proving that the main excretion route of Rg1 was bile excretion. The study also shows that about 88.72% of Rg1 is excreted as a prototype [82]. This result is consistent with the result of the work of Li et al., that is, Rg1 experiences biliary excretion and reaches the small intestine at two different time points, resulting in a bimodal concentration-time curve [80].

#### 2.3.2. Anti-Ischemic Effects and Mechanisms of Rg1

Rg1 shows specific pharmacological effects: promoting cerebral angiogenesis [85], alleviating oxidative stress [86], inhibiting apoptosis [87], decreasing BBB permeability [88], attenuating protein aggregation and inflammatory responses [89, 90], and regulating systemic metabolic alterations [91].

Rg1 was found to ameliorate cerebral ischemic injury in a model of permanent occlusion of both the middle cerebral artery (MCA) and the common carotid artery (CCA) (dMCAO). Compared with the dMCAO group, the Rg1 group exhibited improved neurobehavioral outcomes, reduced brain infarct volumes, enhanced expression of CD31, and increased numbers of BrdU^+^/CD31^+^ microvessels and glial fibrillary acidic protein- (GFAP-) positive vessels in the peri-infarct cortex. In an in vitro experiment, Rg1 notably increased the proliferation, migration, and tube formation of OGD-induced human brain microvascular endothelial (hCMEC/D3) cells. In addition, Rg1 upregulated the expression of VEGF, HIF-1*α*, and PI3K and increased the phosphorylation of AKT and mTOR [85]. These mechanisms might be associated with an ability to promote cerebral angiogenesis by increasing the expression of VEGF via the PI3K/AKT/mTOR signaling pathway after ischemic stroke.

In addition to its roles in regulating oxidative injury, inflammation, and apoptosis, Rg1 was also found to attenuate cell injury in a dose-dependent manner, which was accompanied by the following effects: prolonged nuclear accumulation and enhanced transcriptional activity of Nrf2 and enhanced expression of antioxidant response element- (ARE-) targeted genes [86]; reduced expression of the proapoptotic proteins cleaved caspase-3 and Bax; elevated expression of the antiapoptotic protein Bcl-2; attenuated OGD-induced oxidative stress and suppressed p38/JNK2 phosphorylation [87]; increased BDNF expression in the hippocampal CA1 region; decreased serum levels of IL-1ß, Il-6, and TNF-*α* [89]; and suppressed nuclear translocation of NF-*κ*B and phosphorylation of I*κ*B*α* [90]. These findings were confirmed by the results of different types of ischemia models, such as OGD/R-induced PC12 cells [86], OGD-induced neural stem cells (NSCs) [87], MCAO C57BL/6J mice [89], and MCAO Sprague-Dawley (SD) rats [90].

Furthermore, Rg1 decreases BBB permeability [88]. It also downregulates the expression of protease-activated receptor (PAR)-1 [88], the prototypical PAR isoform, which is activated by thrombin [92], and was shown to mediate thrombin-induced cell death in a model of cerebral ischemia [93].

In summary, Rg1 exhibits anticerebral ischemia activity that may be attributed to its ability to increase VEGF expression via the PI3K/AKT/mTOR signaling pathway [85], alleviate oxidative stress by inhibiting miR-144 activity and subsequently promoting Nrf2/ARE pathway activity [86], increase BDNF expression and downregulate inflammatory cytokine expression [89], inhibit p38/JNK2 phosphorylation in NSCs [87], and downregulate PAR-1 expression [88]. As shown in Table 3, these signaling pathways are involved in promoting angiogenesis [85]; decreasing BBB permeability [88]; and playing antioxidant [86]; anti-inflammatory [89, 90]; and antiapoptotic [87] roles in ischemic brain injury.

### 2.4. Tanshinone IIA (TSA)

**Figure 5:**
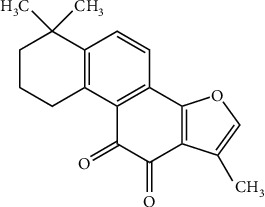
Chemical structural formula of TSA.

Danshen, the dried roots and rhizomes of *Salvia miltiorrhiza*, has been widely used in China to treat cardiovascular and cerebrovascular diseases [94]. Danshen primarily contains varied chemical components, such as tanshinones, salvianolic acids, volatile oils, and inorganic elements. Tanshinones are the main components of the lipid-soluble compounds derived from the roots of Danshen; among the active ingredients of tanshinone, TSA has the highest content [95] (see Figure 5 for the chemical structure). However, due to its poor solubility, the water-soluble sulfonate sodium tanshinone IIA sulfonate (TSA sulfonate) has been used in clinical research and was approved by the China Food and Drug Administration (CFDA) to treat coronary heart disease and ischemic stroke [96].

#### 2.4.1. Pharmacokinetics of TSA

TSA has low water solubility and exposure to first-pass metabolism, so its oral bioavailability is low. Lipid nanocapsules (LNCs) are a nanoplatform that improves oral bioavailability. Ashour et al. enhanced the oral bioavailability of TSA by embedding LNC for the first time. The results showed that the absorption rate and degree of TSA-LNC were significantly higher than those of TSA suspension. In addition, the half-life and average residence time of TSA-LNC increased significantly [97]. Wang et al. combined TSN and TMP into a composite material and prepared oil-in-water nanoemulsions to extend the circulation time in vivo and in vitro and improve the bioavailability of TSN [98].

TSA was rapidly distributed to all kinds of rat tissues after oral administration, and the tissue concentration of TSA decreased in the order of stomach > small intestine > lung > liver > fat > muscle > kidney > spleen > heart > plasma > brain > testis. But the concentration in the brain is low. It may be due to the fact that p-glycoproteins and/or other transporters are located in the BBB.

Bjornsson et al. studied the metabolism of TSA in rat liver microsomal enzymes. The results showed that TSA was rapidly metabolized in rat liver microsomes. The metabolism of TSA changed linearly at incubation time (1~10 min) and protein concentration (0.05~0.4 mg/mL). When the concentration of TSA is greater than 2 *μ*mol/L, its metabolic rate no longer increases with the increase of the substrate concentration but shows a decreasing characteristic [99]. Bi et al. found that ticlopidine (CYP2C19 specific inhibitor) and ketoconazole (CYP3A1 specific inhibitor) can significantly inhibit the metabolism of TSA, indicating that CYP3A1 and CYP2C19 are mainly involved in the metabolism of TSA [100]. The study on the effects of different enzyme inhibitors on drug metabolism provides a basis for mastering the law of drug metabolism.

The primary excretion route of TSA is biliary and fecal excretion, while urinary excretion is the secondary route. After intravenous injection of 60 mg/kg TSA in rats, the recovery rates of TSA within 96 h in urine, feces, and bile were 0.01%, 16.46%, and 12.71%, respectively. Moreover, the kidney accumulation of TSA is lower than that of the lung and liver [101].

#### 2.4.2. Anti-Ischemic Effects and Mechanisms of TSA

TSA sodium injection for clinical application is given by intravenous drip of 80 mg each time. In rats, 4 or 8 mg·kg^−1^ (1/2 or 1 clinical equivalent dose) TSA sodium injection was usually injected intraperitoneally to evaluate its protective effect on ischemic stroke. Accumulating research results have shown that TSA possesses antioxidant, anti-inflammatory, and antiapoptotic properties, which are manifested mainly as a reduced generation of oxidation products [102], inhibition of glial cell activation [95, 103], and decreased in neuronal cell apoptosis [104]. These effects of TSA contribute to suppress the development and progression of cerebral ischemia.

Changes in the biochemistry and physiology of astrocytes and microglia are associated with the secretion of neuroprotective and proinflammatory factors [105, 106]. TSA (4 or 8 mg·kg^−1^ body mass, daily) was found to reduce the intensity of microglial activation, as evidenced by CD11b staining [103], and it relieves astrogliosis, as characterized by the decreased GFAP expression [95]. In addition, the TSA (4 or 8 mg·kg^−1^ body mass, daily) treatment significantly decreased the apoptosis rate of OGD-induced rat neuronal cells, downregulated the expression of Bax, caspase-3, and caspase-8, and upregulated Bal-2 expression [95, 104]. Additionally, Nrf2 is a multipotent transcription factor that can coordinate the antioxidation and detoxification processes through target genes containing an ARE [4, 107]. TSA (25 mg·kg^−1^ body mass, daily) treatment was found to upregulate the expression of Nrf2 mRNA and the content of Nrf2 protein in a nuclear extract. Furthermore, Nrf2 activation by TSA treatment increased the levels of antioxidant enzymes and reduced the generation of oxidation products [102].

The autophagy pathway is the main mechanism for maintaining the balance between the formation and degradation of cellular proteins and damaged organelles, and it greatly impacts cell survival [108, 109]. TSA sulfonate (20 or 40 mg·kg^−1^ body mass, daily) was found to attenuate the MCAO-induced upregulation of autophagy-associated proteins, such as LC3-II, Beclin-1, and Sirt 6 [108], demonstrating that TSA sulfonate can provide remarkable protection against ischemic stroke, possibly via the inhibition of autophagy.

Generally, as shown in Table 4, TSA may inhibit oxidative damage, inflammation, apoptosis, and autophagy in rats/mice with MCAO-induced ischemia [95, 102–104, 108] and in OGD-induced rat primary neuronal cells [104]; these processes are involved in activating the Nrf2-dependent antioxidant response [102], inhibiting glial cell activation [95, 103], and decreasing the expression of apoptosis- and autophagy-associated proteins [95, 104, 108].

### 2.5. Gastrodin (Gas)

**Figure 6:**
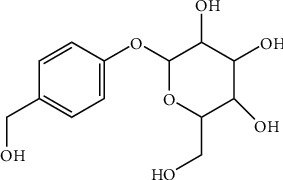
Chemical structural formula of Gas.

*G. elata* Blume, which is called tianma in Chinese, is a renowned herbal plant that has traditionally been used to treat epilepsy, headaches, dizziness, paralysis, convulsions, and other disorders in East Asia for hundreds of years [111]. To date, more than 81 compounds have been isolated from *G. elata* including phenolics, polysaccharides, organic acids, sterols, etc. [112]. Gas (see Figure 6 for the chemical structure) is an extract of dried tubers from *G. elata*, and it is the material basis for its efficacy and is a phenolic compound [113].

#### 2.5.1. Pharmacokinetics of Gas

Gas is absorbed quickly in the body. In rats, Gas could be detected in plasma less than 5 minutes after intragastric (i.g.) administration (100 mg/kg), and the time to reach the highest plasma concentration (*t*_max_) was only 0.42 h [114]. The absolute bioavailability of Gas in rats was more than 80% [115], the plasma protein binding rate was 4.3%, and its aglycone was 69.3% [116]. After intravenous injection of Gas, the plasma concentration-time curve in rats [115, 117] and healthy people [114] accorded with the two-compartment model, while the intragastric administration conformed to the single-compartment model.

Gas is distributed widely after entering the systemic circulation [118]. Jiang et al. measured the distribution and excretion of Gas in the heart, liver, spleen, lung, kidney, brain, and urine of rats. The results showed that the distribution of Gas can be detected in the kidney, liver, and lung, but not in the brain [119]. It is speculated that Gas failed to penetrate the BBB. However, another study confirmed that Gas can enter the brain through the BBB, but the amount of Gas into the brain is less, mainly in the form of aglycone [117, 120].

Gas is rapidly metabolized in the body, and its main metabolite is p-hydroxybenzyl alcohol (the aglycone form of Gas) [121, 122]. Some studies have systematically reported the metabolism and brain pharmacokinetics of Gas in vivo and in vitro. The results showed that Gas metabolized slowly in brain and liver homogenate, rapidly metabolized in renal homogenate, and metabolized to p-hydroxybenzyl alcohol in brain and plasma, but the concentration in brain and plasma decreased rapidly [117]. In recent years, there has been a new understanding of Gas metabolism. Jia et al. identified four metabolites of methyl-d-glucoside, p-hydroxybenzenesulfonic acid, p-methyl-d-glucoside, and p-hydroxy benzaldehyde in rat plasma for the first time [122]. Tang [123], Jiang [119], and Liu [124] studies have shown that parishin can be metabolized into Gas in vivo. Furthermore, the direction of intestinal microorganisms is also related to the study of drug metabolism. For example, Nepal et al. used antibiotic-treated rats to study the effect of intestinal microorganisms on the conversion of Gas to p-hydroxybenzyl alcohol in vivo and in vitro and confirmed the role of intestinal microorganisms in the absorption of Gas into blood [125].

Gas was eliminated quickly. Liu et al. reported that the *t*_1/2_ of rats after i.g. and intravenous (i.v.) of Gas (21 mg·kg^−1^) were 1.13 and 1.30 h, respectively. Ju et al. reported that the distribution *t*_1/2_ of Gas in human plasma was 3.78 h, and the elimination *t*_1/2_ was 6.06 h [114]. The excretion pathway of Gas is mainly excreted from urine by prototype drugs [119], a small amount excreted from bile, and almost none excreted from feces [126]. The metabolism of Gas in mice is affected by enterohepatic circulation [127], but there is no enterohepatic circulation in rats [115].

#### 2.5.2. Anti-Ischemic Effects and Mechanisms of Gas

Gas tablets for clinical application are given by oral administration of 100 mg each time. In rats, 40 mg·kg^−1^ (four times the clinical equivalent dose) Gas tablets were usually injected intraperitoneally to evaluate its protective effect on ischemic stroke. Multiple in vivo experiments have shown that Gas may reduce ischemic injury by inhibiting apoptosis [128–131], Zn^2+^ toxicity [132], inflammation [130, 131], and oxidative damage. First, in a permanent MCAO model established by inducing cerebral ischemic stroke in adult male mice [128, 131] and rats [129, 132] and in a model of subacute-phase cerebral I/R injury in SD rats [130] treated with or without Gas, the infarct volume, neurological score, and histological damage were significantly reduced; the Bax and caspase-3 levels in the ischemic hemisphere were decreased [128–131]; Zn^2+^-induced cell death caused by excessive ROS production was suppressed [132]; the TNF-*α*, IL-1*β*, cyclooxygenase-2, iNOS, and MDA levels were decreased; and the HO-1 and SOD1 expression levels were increased [131] in the Gas-treated groups. These findings indicate that Gas inhibits apoptosis, Zn^2+^ toxicity, inflammation, and oxidative damage in in vivo models of cerebral ischemic injury.

Moreover, treating with Gas was demonstrated to promote the neurogenesis [128] and microvascular regeneration [129] that occurred after ischemic injury by increasing the levels of multiple indicators in a permanent MCAO model, increasing the number of DCX/BrdU double-positive cells [128] and upregulating the expression of VEGF [129].

The studies evaluated above (Table 5) demonstrate that treating with Gas significantly inhibits apoptosis [128–131], Zn^2+^ toxicity [132], inflammation [130, 131], and oxidative damage. Moreover, Gas can promote neurogenesis [128] and microvascular regeneration [129].

### 2.6. Baicalin (BA)

**Figure 7:**
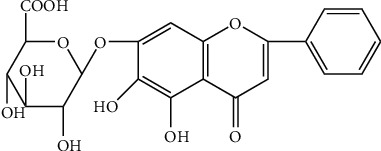
Chemical structural formula of BA.

*Scutellaria baicalensis* is a common herbal medicine that is used to treat bacterial infections of the respiratory and gastrointestinal tracts and a series of inflammatory diseases [133]. BA is a natural flavonoid that is extracted from the dried roots of *Scutellaria baicalensis*, and it exhibits antioxidant, antiapoptotic, and anti-inflammatory biological activities [134–136] (see Figure 7 for the chemical structure).

#### 2.6.1. Pharmacokinetics of BA

The intramolecular hydrogen bond of baicalein (the aglycone form of BA) leads to poor water solubility and low bioavailability after oral administration. However, baicalein is well absorbed by the gastrointestinal tract due to its good lipophilicity [137]. Since baicalein is absorbed better than BA, the conversion of BA to baicalein is a key step in the process of BA absorption [138]. Studies have shown that BA is easily hydrolyzed into baicalein by *β*-glucuronidase derived from intestinal bacteria [139]. Therefore, intestinal bacteria are the key determinant of the conversion of BA to baicalein after oral treatment.

The tissue distribution of BA showed that the concentration of BA was highest in the kidneys [140]. However, another study found that after oral administration of Huang-Lian-Jie-Du-Tang preparation in MCAO rats, the concentration of BA in the lung was higher than that in the kidney or liver. This was consistent with its pharmacological sites of action. Such differences may be caused by different routes of administration, different preparations, or the multiple herbs present in the decoction [141]. Recent studies also have reported that BA could penetrate the BBB and distribute in the brain nucleus [142]. Huang et al. used in vivo microdialysis sampling method coupled with ultraperformance liquid chromatography-tandem mass spectrometry (UPLC-MS/MS) to continuously monitor the BA in rat blood and brain. The results showed that BA could cross the BBB and was detectable in brain dialysate [143].

The metabolism of BA can affect its efficacy and even its toxicity. Zhang et al. identified 23 BA metabolites in rat urine and 26 in rat plasma. Comparing the structures of these metabolites, it can be concluded that the main active metabolic sites are the hydroxyl groups on the ring and the 8- and 4′-positions of BA. It was also found that BA primarily underwent methylation, hydrolysis, hydroxylation, methoxylation, glucuronide conjugation, sulfate conjugation, and their composite reactions [144].

BA is mainly excreted in bile in the form of glucuronides with high biliary excretion [145]. MRP2 is one of the main transporters mediating bile efflux of BA [137]. When BA was injected into the portal vein of MRP2-deficient rats, the biliary excretion of BA decreased significantly, while the content of BA in systemic circulation increased significantly [146]. This highlights the key role of biliary excretion in the pharmacokinetic regulation of BA. Compared with the bile route, the proportion of BA excreted in urine is very small. Lai et al. found that after oral administration of *Scutellaria baicalensis* in male health volunteers, only 7.2% of the dose was excreted in urine in the form of conjugated baicalein. The reason may be due to the remaining proportion of the dose excreted in bile.

#### 2.6.2. Anti-Ischemic Effects and Mechanisms of BA

BA capsules for clinical application are given by oral administration of 500 mg each time. In rats, 100 mg·kg^−1^ (twice the clinical equivalent dose) BA capsules were usually injected intraperitoneally to evaluate its protective effect on ischemic stroke. Accumulating evidence has shown that BA can penetrate the BBB and effectively treat cerebral ischemia [142]. The inhibition of MMP-9 expression and activity is involved in increasing BBB permeability. Rats subjected to MCAO for 4.5 h followed by continuous t-PA infusion (10 mg·kg^−1^) for 0.5 h and then by 19 h of reperfusion exhibited a significantly increased mortality rate and induced hemorrhagic transformation (HT), MMP-9 expression, and apoptotic cell death. However, the results showed that these phenomena were reversed by BA (100 mg·kg^−1^ body mass, daily) [147].

In addition to playing roles in the regulation of oxidative stress and apoptosis, BA can also inactivate mitochondrial succinate dehydrogenase (SDH) to suppress ROS production and promote glutamine synthetase (GS) protein stability to resist oxidative stress under conditions of OGD/R or I/R [148], improve learning and memory deficits induced by global cerebral I/R in gerbils by attenuating the phosphorylation of CaMKII and further preventing hippocampal neuronal apoptosis [149], inhibit apoptosis, and protect neonatal rat brains against hypoxic-ischemic injury by upregulating glutamate transporter 1 via the PI3K/AKT signaling pathway [150].

Hyperglycemia is a risk factor for cerebral I/R injury [151]. In an in vitro experiment, OGD/R induced excessive ROS production, and mitochondrial dynamic impairment was significantly enhanced under high-glucose (HG) conditions. However, in PC12 cells, BA treatment inhibited dynamin-related protein 1 (Drp-1) expression, decreased mitochondrial fission, promoted mitofusin-2 (MFN2) expression, increased Drp-1 phosphorylation on Ser637, and elevated the mitochondrial membrane potential (*Δψ*m) by suppressing ROS production [151]. Furthermore, BA suppressed apoptosis and enhanced mitophagy by regulating mitochondrial functions in an AMPK-dependent manner [151].

Therefore, as shown in Table 6, the pharmacological effects of BA may be attributed to its ability to regulate the expression levels of MMP-9, Drp-1, MFN2, AMPK, and PI3K/AKT and the phosphorylation of Drp-1 on Ser637. These signaling pathways are involved in attenuating BBB disruption [147], regulating mitochondrial functions [151], suppressing apoptosis [149–151], enhancing mitophagy [151], and decreasing oxidative stress [148].

## 3. Chinese Herbal Extracts

### 3.1. Ginkgo Biloba Extract (EGB)

**Figure 8:**
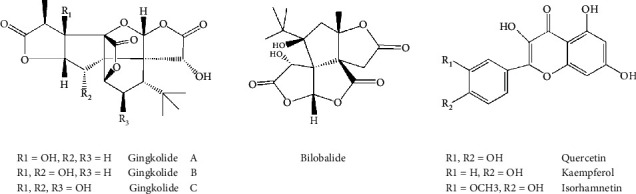
Chemical structural formula for the representative components of ginkgo biloba extract.

EGB is a mixture of various medicinal ingredients extracted from *Ginkgo biloba* leaves, and its main active ingredients are flavonoids, terpene lactones, and organic acids (Figure 8). EGB has pharmacological effects, such as antioxidation, anti-inflammatory, and antiapoptosis activities; it protects mitochondrial function; and it has significant therapeutic effects against strokes, angina, and other cardiovascular and cerebrovascular diseases. EGB is widely used and has been incorporated into various Chinese patent medicines and Chinese medicine injections in clinical practice. Currently, the ginkgo preparations commonly used in clinical practice include Yinxingmihuan oral liquid, Shuxuening injections, and extract of ginkgo biloba leaves injection.

#### 3.1.1. Pharmacokinetics of EGB

One of the insurmountable problems in EGB application is its low bioavailability. Considering the biocompatibility between EGB and starch, Wang et al. loaded EGB onto starch nanospheres (SNP) by nanoprecipitation. It was found that compared with unloaded SNP, the mean sizes of the monodispersed and spherical EGB-loaded SNP were larger. In addition, in artificial gastric and intestinal juices, the EGB s-loaded SNP showed a better sustained release than free EGB [153]. As a new oral drug delivery system, proliposome was used to improve the solubility of active components of EGB. Zheng et al. found that compared with Ginaton, EGB has a significant effect on the absorption, elimination, and bioavailability of flavonoids and terpenoid lactones. The results would be helpful to guide the development of the new oral drug delivery system [154].

Flavonoids are mainly metabolized by the liver and intestinal flora to generate aglycones. Cytochrome oxidase in the liver combines with glucuronic acid to form a conjugate through a biphasic reaction [155]. Moreover, compared with ginkgolides, flavonoids can also inhibit the action of CYP3A enzyme.

Terpene lactones have moderate membrane permeability and are mainly absorbed by passive diffusion. The ester ring in terpene lactones is easily opened in alkaline environment. It is phase I reaction in the liver in vivo, and the metabolism in vitro is similar to that of flavonoids, which is phase II reaction. In addition, terpene lactones can affect the activity of CYP450 enzymes; thus, more studies focus on the relationship between terpene lactones and metabolic enzymes [156].

#### 3.1.2. Anti-Ischemic Effects and Mechanisms of EGB

EGB is a classical herbal product extracted from *Ginkgo biloba* leaves and has a prominent antioxidant role [157].

EGB tablets for clinical application are given by oral administration of 80 mg each time. In rats, 15 mg·kg^−1^ (nearly twice the clinical equivalent dose) EGB tablets were usually administrated intragastrically to evaluate its protective effect on ischemic stroke. Its redox pathway activity was assessed by evaluating the glutathione (GSH), malonaldehyde (MDA), superoxide dismutase (SOD), and NO levels and activities, which revealed that long-term EGB (100 mg·kg^−1^ body mass, daily) administration increased both the GSH and SOD activity, while the levels of MDA and NO were decreased in aged mice on d 7 after MCAO surgery, and the results showed that the antioxidative mechanisms acted by attenuating ERK activation and stimulating PP2A expression [157].

The neurorestorative processes involving angiogenesis, neurogenesis, axon sprouting, and myelin regeneration are essential for long-term neurofunctional recovery after stroke [158]. An investigation focusing on neurovascular restoration and axonal remodeling after ischemic stroke revealed that administering EGB (60 mg·kg^−1^ body mass, daily) for 15 d reduced the infarct volume, alleviated gray and white matter damage, and enhanced the collateral circulation, cerebral perfusion, and axonal remodeling. Importantly, EGB facilitated behavioral recovery and amplified endogenous neurogenesis, and it was accompanied by increases in the p-AKT and p-GSK-3*β* levels and decreases in the p-CRMP2 level [159].

In addition, EGB (15 mg·kg^−1^ body mass, daily) was found to markedly attenuate cerebral infarctions and neurological deficiencies [160] and to inhibit astrocyte activation and neuronal apoptosis [160–163]. The mechanisms underlying these effects involved a reduction in the phosphorylation of STAT3 and JAK2 and a decrease in LCN2 expression [160]. Additionally, EGB inhibited apoptosis [161–163] by preventing caspase-3 cleavage and blocking the extrinsic apoptotic pathway [161], and it reduced the apoptosis rate through the regulation of Bax and Bcl-2 expression [162].

These findings revealed that EGB may exert both neuroprotective and neurorestorative effects, partially by blocking sustained ERK activation and restoring oxidative stress-inhibited PP2A activity [157], promoting endogenous neurogenesis and axonal reorganization by increasing the levels of p-AKT and p-GSK-3*β* and decreasing the expression of p-CRMP2 [159], inhibiting astrogliosis and neuroinflammation via the LCN2-JAK2/STAT3 pathway [160], and inhibiting apoptosis by preventing caspase-3 cleavage and blocking the extrinsic apoptotic pathway [161, 163] (Table 7).

RF: reference; other abbreviations as shown in the literature. (↓): downregulation or inhibition; (↑): upregulation or activation.

## 4. Conclusions and Remarks

The pathophysiology of ischemic stroke provides a range of molecular mechanisms and pathways that are expected to be targeted for intervention [5]. During the early acute phase of ischemic injury, BBB perturbations, free radical production, cerebral edema, and excitatory amino acid toxicity are believed to predominate with key roles. Inflammation and neuronal apoptosis are dominant in the middle phase. During the delayed phase, angiogenesis, axon sprouting, and neurogenesis may provide critical neurovascular substrates for neuronal remodeling [158, 167]. Therefore, the development of novel therapeutic drugs with omnidirectional and multitarget effects that can intervene throughout the ischemic cascade is urgently needed to improve the treatment of ischemic stroke.

Most neuroprotective agents have a good neuroprotective effect in preclinical studies but an unsatisfactory effect in clinical applications. Currently, no recognized neuroprotective agent is on the market. The reason for the failure of calcium channel blockers, such as nimodipine, in cerebral ischemia treatments during clinical trials may be because the therapeutic window for this drug in animal experiments is less than 1.5 h, which is much shorter than the clinical therapeutic window. Previous failures in the development of neuroprotective agents indicate that the action mechanism, therapeutic window, toxicity, pharmacokinetics, and pharmacodynamics of a drug must be studied in detail before a clinical trial is conducted. In addition, the selection of the animal model and the rationality of the clinical trial scheme also affect the results. For example, most patients with clinical strokes are middle-aged and elderly, while adult animals are most often used in animal experiments. In addition, the clinical complications are diverse in stroke patients, but most animal experiments involve single diseases [12].

**Figure 9:**
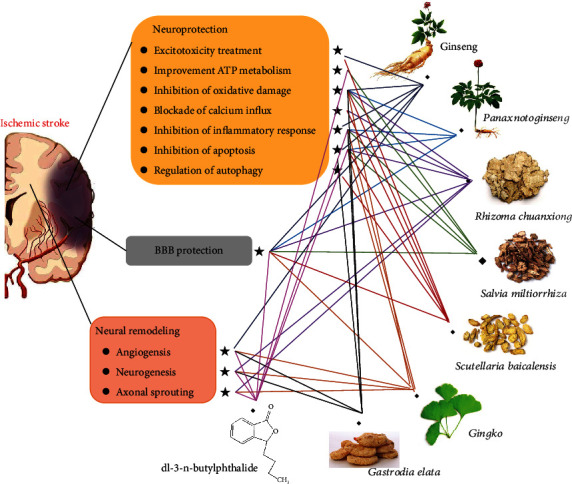
Summary and functional network target analysis of eight types of TCMs that exerts significant anticerebral ischemia effects after ischemic stroke via multiple links across regulatory mechanisms and multitarget effects.

After thousands of years of practice, considerable evidence supporting the use of TCM and natural medicines for the prevention and treatment of ischemic stroke has been collected, and TCM and natural medicines have specific protective effects and few side effects [168]. We found that TCM and its main active constituents have anticerebral ischemia effects that may be achieved partially through their ability to inhibit oxidative stress, apoptosis, and inflammation as well as promote angiogenesis and neurogenesis. The study of single TCMs as stroke treatments has been focused mainly on the role of its active components or effective parts. We summarized a large number of studies to prove that the effective components of TCM, such as TMP, NBP, Rg1, TSA, Gas, and BA, as well as the effective extracts, such as EGB, have preventive and therapeutic effects on strokes (Figure 9). Considering the compatible use of these monomers and screening high-efficiency drugs that affect multiple pathways and targets for stroke treatments can promote and accelerate the research and development of new anti-ischemic drugs.

However, the prevention and treatment of strokes with TCM is still beset with the following problems: first, the composition of TCM compound prescriptions is complex, and a mechanistic analysis remains to be refined; second, the reasonable compatibility of all TCMs in the compound prescription requires in-depth study; third, during stroke treatment, TCM is currently used largely during the stroke recovery stage of or as auxiliary treatment, and few TCMs have been shown to have significant effects on stroke occurrence and treatment [169]. Research on these factors will be the focus of further discussion and analysis.

## Figures and Tables

**Table 1:** Summarized effects and mechanisms of TMP actin on different targets related to ischemic stroke in in vivo and in vitro studies.

Model	Type	Tissue sites	Effects	Mechanisms	RF
p-MCAO in rats OGD/glutamate-induced primary cortical neurons	In vivo In vitro	Ischemic stroke	↓ brain infarction ↓ impairment of behavioral functions ↓ ·OH, O_2_^·-^ and ONOO^−^ expression ↓ Bax expression ↑ Bcl-2 expression	↓ free radicals ↓ calcium overload maintain mitochondrial Function ↑ PI3K/AKT/p-GSK3*β* cell survival pathway	[35]
MCAO in rats Primary cortical neurons	In vivo In vitro	Ischemic stroke	↓ cerebral infarction ↑ neurological function ↑ neurogenesis and Oligodendrogenesis ↑ neuronal differentiation	↑ BDNF/AKT/CREB pathway	[32]
Cerebral I/R in rats	In vivo	Cerebral I/R injury	↓ neurological functional loss ↓ brain edema and BBB permeability ↓ MMP-9 expression ↑ ZO-1, claudin-3 and claudin-5 expression	Restore the integrity and function of BBB ↓ JAK/STAT signaling pathway	[34]
MCAO in rats	In vivo	Ischemic stroke	↑ neurological function ↑ MAP-2 level ↑ spine density of basilar dendrites	↑ dendritic plasticity	[33]
Permanent cerebral ischemia in rats	In vivo	Cerebral ischemia	↓ neuronal loss ↓ macrophage/microglia activation ↓ brain parenchyma infiltrative neutrophils ↓ circulating neutrophils ↓ neutrophil migration ↓ endothelium adhesion ↓ spontaneous NO and stimuli-activated NO production	↑ Nrf2/HO-1 expression ↓ HMGB1/TLR4, AKT, and ERK signaling	[22]
OGD/R-induced BMECs	In vitro	OGD/R injury	↓ platelets adhesion to BMECs ↓ inflammatory cytokines and adhesion molecules expression	↓ P38 MAPK and NF-*κ*B signaling pathways	[23]
Cerebral I/R in rats	In vivo	Cerebral I/R injury	↓ cerebral water content ↓infarction volume ↑ neurological outcomes ↓ blood-brain barrier permeability ↓ TJ proteins junction adhesion molecule-1 and occluding expressions	↓ TJ proteins expression ↓ MMP-9 and AQP4 expressions	[42]
OGD-induced BMECs	In vitro	OGD-induced BMECs injury	↓ apoptosis and permeability ↓ RhoA and Rac1 level ↓ ROS generation ↑ eNOS level ↓ caspase-3 level	↓ rho/ROCK signaling pathway	[36]
MCAO in rats NPCs Primary BMECs	In vivo In vitro	Neuropathological diseases	↑ NPC migration ↑ SDF-1 expression and secretion	↑ PI3K/AKT, PKC, and ERK activity	[40]
H_2_O_2_-induced BMSCs	In vitro	Ischemic stroke	↑ cell viability ↓ cell apoptosis and intracellular ROS generation ↑ Bcl-2 expression ↓ Bax expression	↑ PI3K/AKT and ERK1/2 signaling pathways	[37]
Cerebral I/R in rats OGD-induced HAECs	In vivo In vitro	Cerebral I/R injury	↑ NO, p-AKT/AKT, and p-eNOS/eNOS levels	↑ PI3K/AKT pathway	[43]
MCAO/R in rats	In vivo	Ischemic stroke	↓ neurological deficit ↓ brain water content and infarct size ↓ TNF-*α*, IL-1*β*, MCP-1, ICAM-1 and iNOS concentration ↑ IL-10 concentration ↓ CD11b and ICAM-1 amount	↓ TLR-4 and nuclear NF-*κ*Bp65 expression	[39]
A/R-induced primary hippocampal neurons	In vitro	A/R-induced primary hippocampal neuronal injury	↓ apoptosis rates ↓ JNK kinase MKK4 and MKK7 levels ↓ C-fos, C-jun, and P-JNK levels	↓ apoptosis mediated by JNK/MARK signal pathway	[38]
MCAO in rats	In vivo	Cerebral I/R injury	↓ neurological score ↓ brain infarction and edema levels ↓ BBB permeability	↓ impairment of occludin and claudin-5 ↓ MMP-9 expression and activity	[41]

RF: reference; other abbreviations as shown in the literature. (↓): downregulation or inhibition; (↑): upregulation or activation.

**Table 2:** Summarized effects and mechanisms of NBP on different targets related to ischemic stroke in in vivo and in vitro studies.

Model	Type	Tissue sites	Effects	Mechanisms	RF
Endothelin-1-induced focal cerebral ischemia in rats	In vivo	Focal cerebral ischemia injury	↑ number and length of crossing CST fibers ↑ PSD95 and VGlut-1 levels ↑ number of BrdU^+^/DCX^+^ cells ↓ rho-A^+^, ROCK^+^, Nogo-A^+^ and Nogo-R^+^ cells ↑ behavioral performance	↑ axonal growth ↑ neurogenesis	[53]
p MCAO in rats	In vivo	Cerebral ischemia infarction	↓ glucose and citric acid levels ↑ ATP, ADP, AMP, and GMP metabolism ↑ glutamate, glutamine, aspartate, and N-acetylaspartate levels ↑ glutathione, ascorbic acid, and taurine levels ↓ number of sodium ions ↑ number of potassium ions	↑ ATP metabolism ↑ antioxidant level Maintain sodium-potassium ion balance	[52]
Endothelin-1-induced focal cerebral ischemia in rats	In vivo	Stroke-induced white matter injury	↑ differentiation and maturation of OPCs ↑ length of CST fibers into the denervated hemispheres	↑ Remyelination ↑ BDNF level ↓ NogoA level	[57]
OGD in cortical neurons	In vitro	Ischemic stroke	↑ number of primary and secondary dendrites and of dendritic tips	Regulate dendritic branching ↓ PI3K/AKT Signaling	[54]
p MCAO in mice	In vivo	Cerebral infarction	↓ neurologic function deficits ↓ infarction volume ↓ BBB permeability ↓ MMP-9 expression ↑ Claudin-5 expression	↑ VEGF and GFAP expression ↑ ultrastructure in capillary Endothelial cells of BBBs ↑ Nrf-2/HO-1 signaling pathway	[47]
MCAO in rats	In vivo	Cerebral ischemia	↑ body weight ↓ infarct volume ↑ neurobehavioral outcomes ↑ number of CD31^+^ microvessels ↑ number of CD31^+^/BrdU^+^ proliferating endothelial cells ↑ functional Vascular density ↑ VEGF level ↑ angiopoietin-1 level	↑ angiogenesis ↑ sonic hedgehog expression	[55]
MCAO in mice Photothrombosis-induced permanent cerebral ischemia in mice	In vivo	Ischemic stroke	↓ ICAM-1 level ↓ PAR-1 level ↓ brain infiltration of myeloid cells Preserve BBB integrity ↑ cerebral blood flow	↓ neurovascular inflammation	[48]
t MCAO in mice	In vivo	Cerebral I/R injury	↓ infarct volume ↓ neurological deficit ↓ cerebral edema ↓ BBB permeability	↑ blood flow ↓ BBB dysfunction	[49]
ADP, thrombin, U46619, AA, and collagen-induced platelet activation in human	In vivo	Arterial thrombotic diseases heart attack and stroke	↓ human platelet Aggregation and ATP release PAC-1 binding ↓ TXA2 synthesis ↓ intracellular calcium mobilization	↓ cPLA2-mediated TXA2 synthesis ↓ PDE level ↑ 3,5-cyclic adenosine monophosphate level	[51]
BCCAO in rats	In vivo	Chronic cerebral hypoperfusion	↑ CBF recovery ↑ hemodynamic compensation ↓ astrogliosis ↓ cell apoptosis Protect hippocampal neurons	↑ hemodynamics ↑ CBF recovery ↑ cognitive function	[50]
MCAO in mice	In vivo	Ischemic stroke	↑ functional recovery ↑ number of RECA-1 positive vessels ↑ occludin expression ↑ HIF-1*α* expression ↑ VEGF, notch, and DLL-4 expression	↑ white matter integrity, ↑ occludin expression ↑ HIF-1*α*/VEGF/notch/Dll4 expression	[64]
OGD in SV40-transformed aortic rat endothelial cell line	In vitro	Ischemic injury	↓ endothelial cells injury ↑ PGC-1*α* expression ↑ vascular proliferation	Maintain the endothelial PGC-1*α* expression via regulating eNOS activity	[56]

RF: reference; other abbreviations as shown in the literature. (↓): downregulation or inhibition; (↑): upregulation or activation.

**Table 3:** Summarized effects and mechanisms of Rg1 on different targets related to ischemic stroke in in vivo and in vitro studies.

Model	Type	Tissue sites	Effects	Mechanisms	RF
d MCAO in mice OGD-induced hCMEC/D3 cells	In vivo In vitro	Ischemic stroke	↑ neurobehavioral outcomes ↓ brain infarct volume ↑ CD31 expression ↑ BrdU^+^/CD31^+^ microvessels ↑ GFAP-positive vessels ↑ proliferation, migration, and tube formation of endothelial cells	↑ VEGF, HIF-1*α*, PI3K, p-AKT, and p-mTOR expression ↑ PI3K/AKT/mTOR signaling pathway	[85]
t MCAO in rats OGD/R-induced PC12 cells	In vivo In vitro	I/R-induced neuronal injury	↓ cell injury ↓ MiR-144 activity Prolong Nrf2 nuclear accumulation ↑ Nrf2 transcriptional activity ↑ ARE-targeted genes expression	↓ oxidative stress ↑ Nrf2/ARE Pathway ↓ MiR-144 activity	[86]
MCAO in mice	In vivo	Cerebral I/R injury	↓ infarct volume ↓ neurological deficit scores ↓ IL-1ß, TNF-*α*, and IL-6 contents ↓ Glu and Asp contents	↑ BDNF expression ↓ IL-1ß, IL-6, and TNF-*α* expression ↓ Glu and Asp contents	[89]
OGD-insulted NSCs	In vitro	Ischemic stroke	↓ NSC viability ↓ OGD-induced apoptosis ↓ cleaved caspase-3 and Bax expression ↑ Bcl-2 expression	↓ oxidative stress ↓ p38/JNK2 phosphorylation	[87]
MCAO/reperfusion in rats	In vivo	Cerebral I/R injury	↑ neurobehavioral function ↓ infarct volume ↓ BBB permeability	↓ PAR-1 expression	[88]
MCAO in rats	In vivo	Cerebral I/R injury	↓ infarct volume ↓ neurological deficit ↓ proinflammatory cytokine expressions ↓ proteasomal activity ↓ protein aggregate accumulation	↓ NF-*κ*B nuclear translocation ↓ I*κ*B*α* phosphorylation ↓ ubiquitinated aggregates ↓ inflammatory response	[90]

RF: reference; other abbreviations as shown in the literature. (↓): downregulation or inhibition; (↑): upregulation or activation.

**Table 4:** Summarized effects and mechanisms of TSA on different targets related to ischemic stroke in in vivo and in vitro studies.

Model	Type	Tissue sites	Effects	Mechanisms	RF
Acute ischemic stroke patients	In vivo	Acute ischemic stroke	↑ neurologic functional outcomes ↓ BBB leakage and damage	↓ MMP-9 level	[110]
MCAO in mice	In vivo	Ischemic stroke	↓ neurological scores ↓ infarct volume ↓ cellular apoptosis ↑ Nrf2 mRNA and protein expression ↓ carbonyl protein, nitrotyrosine protein, 8-OHdG, and MDA contents ↑ T-AOC, SOD, CAT, and GSHpx contents	↑ nuclear factor erythroid 2-related factor-dependent antioxidant response	[102]
MCAO in rats	In vivo	Cerebral ischemic injury	↓ infarct size ↓ caspase-3 and caspase-8 expression ↓ GFAP level	↓ caspase-3 and caspase-8 expression ↓ GFAP level ↓ cell inflammation and death extent	[95]
MCAO in rats	In vivo	Cerebral ischemic injury	↑ NeuN level ↑ protein disulfide isomerase and adenosine triphosphatase (Na(+)/K(+)-ATPase) levels ↓ microglial activation	↓ neuronal loss ↑ protein disulfide isomerase level ↑ Na^+^/K^+^-ATPase level ↓ microglial activation	[103]
MCAO/R in mice	In vivo	Cerebral I/R injury	↓ infarct volumes ↓ neurological deficits ↓ brain water contents ↓ infiltration of macrophages and neutrophils ↓ numbers of macrophages, T cells, and B cells	↓ LC3-II, Beclin-1 and Sirt 6 ↓ autophagy and inflammatory activity	[108]
MCAO in rats OGD-induced rat primary neuronal cells	In vivo In vitro	Cerebral infarction	↓ cerebral Infarct volume, cerebral edema, and neurological deficits score ↓ cell apoptosis ↓ IL-6, TNF-*α*, and CRP levels ↑ cell viability ↓ cell apoptosis ratio ↓ Bax level ↑ Bcl-2 level	↓ IL-6, TNF-*α*, and CRP levels ↓ Bax level ↑ Bcl-2 level ↓ neuronal cell apoptosis and inflammatory response	[104]

RF: reference; other abbreviations as shown in the literature. (↓): downregulation or inhibition; (↑): upregulation or activation.

**Table 5:** Summarized effects and mechanisms of Gas on different targets related to ischemic stroke in in vivo studies.

Model	Type	Tissue sites	Effects	Mechanisms	RF
p MCAO in mice	In vivo	Cerebral ischemic stroke	↑ neural function ↓ infarct volume and apoptosis ↑ number of DCX/BrdU double-positive cells	Restore the Wnt/*β*-catenin signaling pathway ↑ neurogenesis	[128]
p MCAO in rats	In vivo	Acute cerebral infarction	↓ neurological score ↓ CRP and IL-1*β* levels ↑ Bcl-2 expression ↓ BAX and caspase-3 expression	↓ apoptosis ↑ VEGF expression ↑ microvascular regeneration	[129]
t MCAO in rats	In vivo	Postischemic brain	Induced up-regulation and nuclear translocation of Nrf2 ↑ antioxidative genes expression, such as HO-1 and GCLM, in astrocytes ↓ Zn^2+^-induced cell death	↓ p67 expression and PAR formation in astrocytes ↓ Zn^2+^-toxicity and oxidative effects	[132]
Subacute phase cerebral I/R injury in rats	In vivo	Subacute phase cerebral I/R injury	↓ I/R-induced disability and histological damage ↓ neuronal apoptosis ↓ interleukin-1*β*, cyclooxygenase-2, inducible nitric oxide synthase, and cleaved caspase-3 levels	↓ interleukin-1*β*, cyclooxygenase-2, inducible nitric oxide synthase, and cleaved caspase-3 levels ↓ inflammation and apoptosis	[130]
MCAO in mice	In vivo	Cerebral ischemic damage	↓ neuronal injury and neurobehavioral deficient ↓ caspase-3 and Bax expression ↑ Bcl-2 expression ↓ MDA content, TNF-*α* and IL-1*β* expression ↑ SOD activity, HO-1, and SOD1 expression	↑ AKT/Nrf2 pathway	[131]

RF: reference; other abbreviations as shown in the literature. (↓): downregulation or inhibition; (↑): upregulation or activation.

**Table 6:** Summarized effects and mechanisms of BA on different targets related to ischemic stroke in in vivo and in vitro studies.

Model	Type	Tissue sites	Effects	Mechanisms	RF
MCAO in rats	In vivo	Ischemic stroke	↓ mortality rates ↓ t-PA-mediated BBB disruption ↓ hemorrhagic transformation	Scavenge peroxynitrite ↓ MMP-9 expression and activity ↓ Peroxynitrite-mediated MMP-9 activation	[147]
STZ injection aggravated the brain damage induced by MCAO surgery OGD/REP-induced P12 cells	In vivo In vitro	Cerebral I/R injury	↓ blood glucose, neurological deficit, and infarct volume ↓ ROS production and Drp-1 expression ↓ mitochondrial fission ↑ mitofusin-2 (MFN2) generation ↑ Drp-1 Ser637 phosphorylation ↑ mitochondrial membrane potential	↓ cell apoptosis ↑ mitophagy Regulating mitochondrial functions in an AMPK-dependent manner	[151]
Global cerebral I/R in gerbil OGD-induced SH-SY5Y cells	In vivo In vitro	Ischemia-induced memory impairment	↑ learning and memory impairment ↓ intracellular calcium concentration ↓ phosphorylation level of CaMKII ↓ caspase-3 and Bax expression ↑ Bcl-2 expression	↓ phosphorylation level of CaMKII ↓ hippocampal neuronal apoptosis	[149]
Hypoxic-ischemic encephalopathy in rats	In vivo	Hypoxic-ischemic injury	↓ cerebral infarct volume ↓ neuronal loss ↓ apoptosis ↑ p-AKT and glutamate transporter 1 expression	↑ glutamate transporter 1 via the PI3K/AKT signaling pathway	[150]
MCAO in rats	In vivo	Cerebral ischemic injury	↓ neurological deficits ↓ infarct volume ↑ NeuN, GFAP, and progesterone receptor expression	↑ progesterone and adrenocorticotropic hormone expression	[152]
MCAO/R in rats OGD/R-induced primary astrocytes	In vivo In vitro	Acute ischemic stroke	↓ SDH level ↓ glutamine synthetase loss ↓ ROS production	↓ ROS production ↓ excitotoxicity ↑ glutamate disposal ↓ oxidative stress	[148]

RF: reference; other abbreviations as shown in the literature. (↓): downregulation or inhibition; (↑): upregulation or activation.

**Table 7:** Summarized effects and mechanisms of EBG on different targets related to ischemic stroke in in vivo and in vitro studies.

Model	Type	Tissue sites	Effects	Mechanisms	RF
Microsphere-embolized rats OGD-induced human astrocytes	In vivo In vitro	Ischemic brain stroke	↓ cerebral infarction ↓ neuronal apoptosis ↓ inflammatory cytokine level ↓ neurological deficiencies ↓ astrocyte activation	↓ phosphorylation of STAT3 and JAK2 ↓ LCN2 expression ↓ STAT3 activation ↓ neuroinflammation via the LCN2-JAK2/STAT3 pathway	[160]
MCAO in rats	In vivo	Embolic stroke	↓ infarct volume ↓ gray and white matter damage ↑ collateral circulation ↑ cerebral perfusion ↑ axonal remodeling ↑ behavioral recovery ↑ neurovascular restoration ↑ endogenous neurogenesis	↑ p-AKT, p-GSK-3*β* ↓ p-CRMP2 expression ↑ GAP-43 level ↓ NogoA and NgR levels	[159]
p MCAO in rats	In vivo	Focal ischemic stroke	↑ motor function ↓ neurological scores ↑ latency in rotarod test, walk speed, and the body rotation ↓ stride time and the left posterior swing length in gait	—	[164]
p MCAO in ovariectomized female mice	In vivo	Ischemic brain damage	↑ neurogenesis ↑ androgen receptor expression ↑ grip strength ↓ neurological deficits	↓ apoptosis by preventing caspase-3 cleavage and blocking the extrinsic apoptotic pathway	[161]
OGD/R-induced rat cortical capillary endothelial cell-astrocyte-neuron network	In vitro	Ischemic stroke	↑ neuron cell viability ↓ cell injury ↓ cell apoptotic rate	↑ transendothelial electrical resistance of capillary endothelial monolayers ↓ endothelial permeability coefficients for sodium fluorescein ↑ ZO-1 and occludin levels	[162]
MCAO in rats	In vivo	Acute cerebral infarction	↓ caspase-3 and Bax mRNA and protein levels ↑ Bcl-2 mRNA and protein levels	↓ apoptosis ↓ caspase-3 and Bax mRNA and protein levels ↑ Bcl-2 mRNA And protein levels	[163]
MCAO in aged mice	In vivo	Cerebral-ischemia-induced neuronal damage	↓ infarct volumes and brain edema ↓ oxidative stress	↓ ERK activation ↑ phosphatase PP2A	[157]
Cerebral ischemia-reperfusion injury in mice	In vivo	Cerebral ischemia-reperfusion injury	↓ cerebral infarct size, edema, and neurological deficit score	↓ TWEAK-Fn14 axis	[165]
MCAO in mice	In vivo	Subacute stroke	↑ survival rate, neurological and motor functions ↑ histopathological changes ↓ cerebral infarction ↓ edema volume	↓ G-CSF, E-selectin and MAC-1 levels ↓ granulocyte adhesion and diapedesis	[166]
